# School Administrator and Food Vendor Perspectives on Stocking and Promoting Healthier Offerings in Indonesian Primary Schools: Findings from a Pilot Study

**DOI:** 10.3390/ijerph23010101

**Published:** 2026-01-12

**Authors:** Esther M. Nguyen, Hamam Hadi, Emma C. Lewis, Madelyn Sijangga, Herwinda Kusuma Rahayu, Muhammad Evan Takamitsu Kurniawan, Joel Gittelsohn

**Affiliations:** 1 Human Nutrition, Department of International Health, Johns Hopkins Bloomberg School of Public Health, Baltimore, MD 21205, USA; 2 Penn Medicine, University of Pennsylvania Health System, Philadelphia, PA 19104, USA; 3 Graduate School of Public Health, Faculty of Medicine and Health Sciences, Alma Ata University, Yogyakarta 55183, Indonesia; 4 Alma Ata Center for Healthy Life and Foods (ACHEAF), Alma Ata University, Yogyakarta 55183, Indonesia; 5 Institute for Advancing Health Through Agriculture, Texas A&M AgriLife, Texas A&M University System, Dallas, TX 75252, USA; 6 Boston Children’s Hospital, Boston, MA 02115, USA; 7 Department of Nutrition, Faculty of Medicine and Health Sciences, Alma Ata University, Yogyakarta 55183, Indonesia

**Keywords:** childhood obesity, Indonesia, school canteens, school food environment, multiple methods

## Abstract

**Highlights:**

**Public health relevance—How does this work relate to a public health issue?**

- Childhood overweight and obesity is an increasing public health concern in Indonesia, and school canteens represent a primary food environment for many school-aged children.
- Decisions made by canteen owners and school administrators directly shape the nutritional quality of foods and beverages available to children during the school day.

**Public health significance—Why is this work of significance to public health?**

- This pilot study provides novel insights into canteen owners’ and school administrators’ perceptions of the acceptability and feasibility of stocking healthier, locally available foods and beverages.
- Children’s taste preferences, affordability, visual appeal, perceived nutrition, and profitability emerged as key factors influencing canteen stocking decisions in both urban and rural primary schools.

**Public health implications—What are the key implications or messages for practitioners, policy makers and/or researchers in public health?**

- Future strategies to improve school food environments should account for children’s preferences as well as the economic and operational constraints faced by canteen operators.
- Introducing healthier options that align with existing taste preferences and canteen practices may support more feasible, acceptable, and sustainable school-based nutrition interventions.

**Abstract:**

Childhood overweight and obesity is a growing public health challenge in Indonesia, affecting approximately one in five school-aged children. Because children spend substantial time at school and frequently obtain meals and snacks from on-site canteens, these settings represent an important opportunity for nutrition-focused interventions. As an initial step towards understanding factors influencing canteen stocking decisions, we assessed perceived taste, acceptability, and feasibility of healthier local foods and beverages from the perspectives of canteen owners and school administrators *(n* = 10) across five primary schools (*n* = 2 urban, *n* = 3 rural) in Magelang, Indonesia. Participants completed in-person taste tests of selected food and beverage options and participated in in-depth interviews exploring drivers of stocking decisions. *IndoMilk* (multi-cereal, reduced-sugar dairy beverage) received the most favorable taste ratings and was perceived as the most feasible option to sell, followed by *sate telur puyuh* (braised quail eggs) and *sate buah* (fresh fruit skewers). In contrast, *gethuk* (cassava/coconut cake) and *polo pendem* (steamed tubers with boiled peanuts) were viewed as less appealing to children and unlikely to be sold. Participants identified children’s taste preferences, affordability, visual appeal, and profitability as key considerations influencing stocking decisions, while perceptions of nutrition varied. Findings from this pilot study highlight contextual factors shaping school canteen food environments and may inform future interventions aimed at introducing healthier options while accounting for children’s preferences and canteen operational constraints.

## 1. Introduction

Childhood overweight and obesity is an escalating public health challenge in Indonesia, where one-fifth of school-aged children are overweight and 14.8% of children have obesity [1]. Globally, the prevalence of childhood overweight and obesity doubled between 1996 and 2016, yet in Indonesia, it increased fourfold over the same period [2]. Excess weight in childhood is linked to numerous short- and long-term health consequences, including increased morbidity and premature mortality [3]. Diets high in fat, sugar, and salt, and low in fruit and vegetables, are major contributors to this burden [1,4]. School food environments are especially influential, as Indonesian children spend nearly one-third of their day in school and obtain many of their daily meals and snacks from school canteens or other nearby vendors [4,5,6].

School canteens are designated food stalls operating on school grounds that sell a variety of items, ranging from meals such as rice with meatballs or fried chicken, to deep-fried snacks, packaged foods, and sugar-sweetened beverages [4,7,8]. Although fruit is sometimes available, it is often prepared with syrup, chocolate, and sprinkles to increase visual appeal [4]. As a result, the majority of foods offered are energy-dense, processed, and high in sugar, salt, and fat [4,5,8]. These offerings are primarily determined by the independent owners who operate canteens under the supervision of a manager. While managers, who tend to be a school administrator or teacher, oversee menu coordination and establish general selection criteria, owners ultimately decide which foods to sell and how they are prepared [4,9]. Together, these actors play a pivotal role in shaping the nutritional quality of school children’s daily diets, making their perspectives essential for informing effective interventions to improve school food environments.

Recently, there has been increasing national and international attention to improving school food environments in Indonesia. In May 2025, the United Nations Children’s Fund’s (UNICEF) published a national assessment of 268 primary schools across Java, Sulawesi, and Papua which revealed serious gaps, including limited healthy food options and weak nutrition education, and called for stronger measures to improve school nutrition environments [10]. Building on these findings, UNICEF developed the Nutrition Environment Assessment Toolkit for Schools (NEAT-S) to help “accelerate the implementation of key national food environment policies, including specific measures to promote healthier school nutrition environments,” reinforcing the mounting governmental and institutional emphasis on school-based nutrition interventions [10].

Nevertheless, school canteens continue to prioritize processed, energy-dense products. Contributing factors include children’s preference for such foods, lower profit margins on healthier items purchased in small quantities, competition from low-cost street vendors, limited storage capacity, and weak enforcement of school nutrition policies [2,9,10,11,12]. For instance, a situation analysis in Jakarta revealed that canteen owners often store leftover foods in unsuitable spaces, such as in stairwells or near toilets, making it financially prudent to stock only items that sell quickly [13]. To date, government initiatives to improve school nutrition have been undermined by vague guidelines and poor enforcement, resulting in limited adoption [4,5,14]. These challenges demonstrate the need to better understand how canteen operators navigate child preferences, cost considerations, and operational constraints when selecting foods to offer. Although not guided by a single formal framework, this study aligns with ecological and school food–environment perspectives that recognize the multilevel influences shaping food availability in canteen settings.

Canteens therefore represent a critical yet underutilized avenue for childhood obesity prevention. However, little is known about the barriers and facilitators that shape the complex stocking decisions of canteen owners and managers. Understanding their options, constraints, and decision-making processes is critical for designing effective school-based nutrition interventions. To address this gap, we conducted formative research in urban and rural primary school canteens located in Magelang, Indonesia, to explore the following questions:

1. Which healthier food and beverage options are preferred by canteen owners and school managers for selling to children, based on taste testing?
2. What barriers and facilitating factors influence decisions to stock these healthier options?

## 2. Materials and Methods

### 2.1. Setting and Participants

This pilot study was conducted in five primary schools (*n* = 3 urban, *n* = 2 rural) located in Magelang, Central Java, Indonesia, serving children aged 5–11 years. Participants (*n* = 10 total) included canteen owners (*n* = 5) and school staff administrators (*n* = 5) who supervised canteens in a managerial role. Schools, canteen owners, and managers were recruited through convenience sampling based on existing collaborations with local researchers from Alma Ata University. Canteen owners and managers were selected because they oversee food procurement, preparation, pricing, and sales, positioning their perspectives as essential for understanding the operational realities and feasibility of implementing healthier foods. Ethical approval was provided by the Alma Ala University Institutional Review Board (KE/AA/XII/10111310/EC/2023) in agreement with the Johns Hopkins University Institutional Review Board. Informed consent was obtained from all participants, and verbal assent was provided prior to data collection. Permission to conduct the study was granted by each school principal.

### 2.2. Data Collection

Data collection occurred in two sequential phases: (1) taste tests and (2) in-depth interviews (IDIs). Each participant completed the phases independently. All activities were conducted in Bahasa Indonesia, then translated and transcribed into English by the research team for analysis.

**Phase 1: Taste tests**. Foods and beverages were selected by the research team based on nutritional content, cultural relevance, availability, and appropriateness for the local setting. These five items were specifically selected because they reflect commonly consumed, culturally familiar, and nutritionally dense alternatives that could be assessed for acceptance. Items classified as “healthier” were lower in sugar, minimally processed, and higher in macro- and micronutrient content. Five items were selected: (1) *sate buah* (various fresh fruits including papaya, pineapple, watermelon, cantaloupe, and honeydew prepared on a skewer); (2) *sate telur puyuh* (braised quail eggs); (3) *Chocolate IndoMilk* (multi-cereal dairy beverage with reduced sugar); (4) *polo pendem* (various steamed tubers and boiled peanuts); and (5) *gethuk* (traditional cassava and coconut cake snack) [15,16].

A 3-point facial hedonic scale, adapted from a validated 5-point version [11,12,17], was used to assess perceived taste acceptability. This simplified scale included three animated faces representing “dislike”, “neither dislike nor like”, and “like” [11,12,17]. The faces were intended to be simple and easy to interpret, with labels in Bahasa underneath. The 3-point scale was selected to support rapid, low-burden formative assessment and to facilitate clear interpretation across participants with diverse backgrounds, rather than to provide fine-grained sensory discrimination. For each item, participants rated taste using the scale and answered a follow-up question framed from the perspective of a typical schoolchild: “*Would you buy this for lunch? [Yes or No]*”. Although adult participants completed the taste tests, the scale was used to capture perceptions of child-relevant acceptability and feasibility within a school canteen context. Taste test sessions lasted approximately 20–25 min.

**Phase 2: In-depth interviews.** Immediately following the taste tests, semi-structured IDIs were conducted by a locally trained researcher and a registered dietician (RD) local to Yogyakarta and a native Bahasa speaker. Interviews lasted approximately 30–35 min and explored: (a) perceived barriers and facilitators to stocking and selling healthier foods, (b) items most frequently purchased by children, (c) logistical considerations related to school schedules, and (d) participant reflections on the taste test. Interview guides were adapted from previous studies conducted in this setting [5,18]. Reflexivity was addressed through ongoing discussion among the research team regarding how researchers’ disciplinary training, professional roles, and positionality may have influenced data collection and interpretation. Data collection was led by locally trained researchers fluent in Bahasa Indonesia and familiar with the school and canteen context, which supported rapport and contextual sensitivity. Interpretation of findings was conducted collaboratively with international team members, allowing for triangulation of perspectives and reflection on how local and external viewpoints shaped analytic decisions.

### 2.3. Data Analyses

Quantitative data from taste tests were analyzed descriptively in Microsoft Excel, with percentages calculated for hedonic ratings and purchase intentions.

IDI audio recordings were analyzed thematically using the framework method to generate key themes and identify similarities and differences [19,20,21]. Recordings were first transcribed verbatim in Bahasa and translated into English. Two bilingual team members (H.K.R., M.E.T.K.) independently cross-checked each transcript, and any discrepancies in translation were discussed and resolved collaboratively to ensure accuracy.

For qualitative analysis, transcripts were read repeatedly for familiarization by the first author (E.M.N.), who conducted the initial inductive coding and developed a codebook. The framework and coded segments were then reviewed by an experienced qualitative researcher to assess the clarity, coherence, and consistency of the coding. Any interpretive differences or concerns raised during this expert review were discussed and resolved through consensus. Discrepancies were discussed and resolved collaboratively to ensure that translations accurately represented participant responses. Coding was initially conducted in Microsoft Word and Excel, and later organized and refined in ATLAS.ti version 24.1.1 (Mac version). Emerging themes and subthemes were identified through iterative comparison across transcripts and are reported below.

Although the sample size was small, recurring patterns across interviews indicated adequate information power for the formative aims of this pilot study, while acknowledging that full thematic saturation was beyond scope.

## 3. Results

### 3.1. Taste Perceptions of Healthier Food Options

Perceptions of the tested foods varied by participant role (canteen owner vs. manager) (Table 1). Among the five items evaluated, *IndoMilk* (fortified reduced-sugar dairy drink) received the most favorable ratings, with 9 of 10 participants (90%) indicating they liked the product. *Sate telur puyuh* (quail egg skewers) was liked by 8 of 10 participants (80%), while *sate buah* (fruit skewers) was liked by 7 of 10 participants (70%). In contrast, *gethuk* (cassava snack) received favorable ratings from only 4 of 10 participants (40%), and *polo pendem* (tubers and peanuts) received no favorable ratings.

When asked whether they would sell each item for lunch, owners and managers reported similar responses (Table 1). Most participants (80%) indicated they would be willing to sell *IndoMilk*, while fewer than half reported willingness to sell *sate telur puyuh* (40%) or *gethuk* (40%). Seventy percent indicated willingness to sell *sate buah,* and no participants reported willingness to sell *polo pendem*. Participants attributed willingness to sell *IndoMilk* to affordability, perceived nutritional value, and anticipated demand among children, while concerns about price and children’s preference for sweeter flavors were raised by those unwilling. For *sate buah,* participants noted children’s preference for sweet foods but also cited cost, spoilage, and the need for added ingredients to increase appeal. Participants who were unwilling to sell *sate telur puyuh* and *gethuk* cited portion size relative to price, preparation time, and limited appeal to children. All participants indicated they would not sell *polo pendem*, explaining that children tend to dislike the item, consume it frequently at home, and find its preparation unappealing.

### 3.2. Reported Factors Influencing Stocking Decisions in Canteens

Participants described several factors influencing their decisions about which foods to stock. Most participants (70%) identified children’s preferences as a primary determinant of purchasing behavior. Cost was also frequently cited, with 70% reporting that children’s limited purchasing power constrained which items could be sold.

Perspectives on nutrition varied. Half of participants (50%) stated that nutritional considerations influenced stocking decisions, while others reported that nutrition was not a primary concern when selecting foods and beverages. Participants also described visual appeal as influential, with 40% noting that children were more attracted to sweeter foods and items with visually appealing presentation, such as fruit covered in chocolate or sprinkles.

Participants’ descriptions of nutrition differed across interviews. Some referred to nutrient content or food quality, while others described nutrition in terms of satiety or feeling full. Finally, 30% of participants identified profitability as an important factor, noting that some foods generate higher profit than others.

### 3.3. Overarching Key Themes

Analysis of the interview data identified five overarching themes influencing canteen food offerings: (1) children’s taste preferences, (2) affordability, (3) nutrition, (4) visual appeal, and (5) profitability (Table 2).

**Theme 1. Children’s taste preferences.** Participants consistently emphasized children’s preferences as a primary influence on stocking decisions. Both canteen owners and managers described relying on direct feedback from children as well as observed purchasing patterns to guide food selection. Several participants noted that children lose interest in foods offered repeatedly, underscoring the importance of menu variety to maintain sales.

**Theme 2. Affordability.** Affordability emerged as a key consideration, with participants frequently referencing children’s daily allowances when determining which items to stock and how to price them. Decisions about portion size were often adjusted to balance cost and perceived value, reflecting efforts to align offerings with children’s purchasing power.

**Theme 3. Nutrition.** Perspectives on nutrition varied across participants. Some owners and managers described prioritizing foods they perceived as nutritious or safe for children, while others indicated that nutritional considerations played a limited role in stocking decisions.

**Theme 4. Visual appeal.** Visual appeal was described as an important factor shaping children’s purchasing decisions. Participants noted that foods perceived as colorful, sweet, or attractively presented were more appealing to children, whereas items lacking visual interest were less likely to sell.

**Theme 5. Profitability.** Profitability was identified as a practical driver of canteen operations. Participants highlighted the need to stock items with sufficient profit margins and described pricing strategies that prioritized financial viability, often taking precedence over nutritional considerations.

## 4. Discussion

To our knowledge, this is one of the first studies to examine canteen owners’ and managers’ perceptions and decision-making around stocking healthier foods and beverages for Indonesian primary schoolchildren. Findings from this pilot study suggest that *IndoMilk* (fortified reduced-sugar dairy drink) was perceived as the most appealing and feasible option to sell, followed by *sate telur puyuh* (quail egg skewers) and *sate buah* (fruit skewers). Although *sate telur puyuh* was generally liked, participants raised feasibility concerns related to children’s daily allowances and canteen profit margins. In contrast, *polo pendem* (tubers and peanuts) and *gethuk* (cassava snack) were consistently perceived as unappealing and unlikely to sell. Across interviews, children’s taste preference, affordability, nutritional value, visual appeal, and profitability emerged as salient considerations shaping stocking decisions.

These findings align with prior research from Seoul, Korea, showing that children tend to prefer sweeter-tasting foods and avoid bitter flavors [22]. Such preferences may reflect not only sensory taste but also broader dietary exposure and familiarity [22]. Collectively, this evidence highlights how children’s actual and perceived preferences influence vendors’ perceptions of marketability and sustainability within school environments. These findings underscore the importance of interventions that acknowledge existing taste preferences while gradually encouraging acceptance of healthier options, such as through repeated exposure and nutrition education.

Cost emerged as a major driver of canteen stocking decisions. Prior research indicates that most (81.5%) Indonesian primary schoolchildren receive limited daily allowances, typically ranging between 1000.00–5000.00 IDR ($0.06–$0.30 USD), which may direct purchasing towards inexpensive, energy-dense foods [4,5]. Although school canteen prices are generally low, healthier options—particularly fruits and vegetables—remain less available, in part due to perceptions of reduced value for money [4]. Consistent with this evidence, participants in the present study reported that children often purchase larger quantities of low-cost items rather than smaller portions of more expensive, nutrient-dense alternatives, reinforcing affordability as a key constraint on stocking healthier options.

Nutrition-related considerations also influenced stocking decisions, though participants’ interpretations of what constituted “nutritious” foods varied considerably. While some owners and managers prioritized foods they believed were safe, filling, or health-promoting, others indicated that nutrition played a limited role in food selection. These perceptions did not always align with national dietary guidelines, a pattern observed in prior research from Indonesian university and school canteens [8,22,23,24,25]. Together, these findings suggest that economic and operational constraints may limit the prioritization of nutrition, even when healthier options are recognized.

Perceived visual and sensory appeal also emerged as a key driver of canteen stocking decisions. In this study, healthier foods were often viewed as less attractive to children, consistent with findings from other contexts where students associate healthier items with poorer taste or reduced appeal [24,26]. In contrast, processed pre-packaged items such as instant noodles and powdered sugary drinks tend to be inexpensive, visually appealing, palatable, and easy to prepare [3]. Canteen owners in other settings have similarly reported difficulties complying with healthy canteen guidelines due to concerns about profitability, perishability of fruits and vegetables, and entrenched student eating habits [24]. These patterns underscore the importance of early interventions, ideally beginning at home or in primary school, to help shape children’s preferences before less healthy habits become established.

Taken together, the taste test findings and interview themes provide formative insights that may inform future programs and policies aimed at promoting healthier, appealing, and feasible options within Indonesian school canteens. Participants emphasized that children’s preferences strongly influence purchasing behavior and, in turn, canteen stocking decisions. Healthier options may therefore need to align with familiar taste profiles and be introduced incrementally, alongside existing offerings. Visual appeal also emerged as influential, suggesting opportunities to improve the presentation or placement of healthier foods within the practical constraints of canteen operations.

Building on these findings, several canteen-level strategies could support healthier school food environments. Training or practical toolkits developed by registered dietitians may assist vendors in preparing healthier versions of popular foods, while incentives for stocking lower-sugar or lower-fat products could encourage adoption if financially feasible. Training could also incorporate components related to nutrition education and literacy, given the variability in how nutrition was understood among participants in this study. At the policy level, clearer and more enforceable canteen guidelines, along with vendor training, routine monitoring, and procurement support for affordable healthy ingredients, may help sustain improvements across diverse school settings.

These findings are also worth considering within the context of Indonesia’s Nutritious Meals Program (Makan Bergizi Gratis; MGB), recently launched in January 2025 to address child malnutrition [27]. While this study did not evaluate MGB directly, participants’ perspectives highlight contextual factors, such as feasibility, affordability, and perceptions of nutrition, that may be relevant to future implementation efforts. Because MGB aims to provide nutritionally balanced meals alongside nutrition education, school canteens may represent a natural implementation partner. Integrating canteen owners into MGB could support food safety practices, provide structured nutrition training, and improve the overall healthfulness of foods available in schools. Leveraging this existing national infrastructure may accelerate the adoption of healthier, affordable, and visually appealing food options.

Several limitations should be acknowledged. The small, convenience-based sample and short data collection period limit generalizability. As a pilot study, the intent was to generate preliminary insights rather than population-level estimates. In addition, approvals were not obtained to conduct taste tests directly with children, highlighting an important area for future research. While this study focused on canteen owners and managers, their perspectives provide essential context for understanding the feasibility of introducing healthier options in school food environments.

## 5. Conclusions

School canteens are a primary source of meals and snacks for Indonesian primary schoolchildren and represent a critical leverage point for childhood obesity prevention. This pilot study provides early insights into canteen owners’ and managers’ perceptions of stocking healthier food and beverage options. Incorporating the views of these key stakeholders is essential for designing acceptable and contextually feasible improvements to the school food environment. Future research should build on these findings by incorporating children’s perspectives and examining economic and cultural factors shaping food choices across a broader range of school settings. Policy efforts may benefit from strengthening nutrition and food safety standards and their implementation, while remaining attentive to affordability, taste preferences, and profitability. Multisectoral approaches that balance these considerations may help support the availability and appeal of healthier foods within Indonesian school canteens.

## Figures and Tables

**Table 1:** Taste test ratings and perceptions of five healthier items (*n* = 10).

Food/Beverage	Dislike	Neither Dislike nor Like	Like	*“Would You Sell This for Lunch?”*	
				No	Yes
*IndoMilk Rendah Gula* (Reduced Sugar Chocolate Milk)	10%	0%	90%	20%	80%
Owner	10%	0%	40%	10%	40%
Manager	0%	0%	50%	10%	40%
*Sate Telur Puyuh* (Quail Egg Skewer)	0%	20%	80%	60%	40%
Owner	0%	10%	40%	30%	20%
Manager	0%	10%	40%	30%	20%
*Sate Buah* (Fruit Skewer)	10%	30%	70%	30%	70%
Owner	0%	10%	40%	10%	40%
Manager	0%	20%	30%	20%	30%
*Gethuk* (Cassava Snack)	20%	40%	40%	60%	40%
Owner	10%	20%	20%	20%	30%
Manager	10%	20%	20%	40%	10%
*Polo Pendem* (Various Tubers)	90%	10%	0%	100%	0%
Owner	50%	0%	0%	50%	0%
Manager	40%	10%	0%	50%	0%

**Table 2:** Key themes and relevant quotes depicting drivers of canteen stocking.

Theme	Quotes from Canteen Owners	Quotes from Canteen Managers
Children’s Taste Preferences	“Interviewer: Yes, what are the overall considerations? Participant: Child’s favorite aspect first, their preference” School B, Female “We choose the foods according to children’s preferences” School A, Female	“I will consider children’s favorites…But if we sell the same product every day they will get bored.” School D, Female Interviewer: “Next, how about the consideration factor of selling new products? Participant: What are the children’s preferences in food? We ask the students what they are interested in” School B, Male
Affordability	“We sell something that also suits a child’s pocket money, not something expensive.” School D, Female “The main factor is that the price must be affordable.” School E, Female Interviewer: “How do you determine prices in the canteen?” Participant: “Affordability for children, the average pocket money is Rp 5000.” School C, Female	“Yes, if the fruit is expensive, cut it into small pieces, the children think “oh, only a little amount”. They like big size food but with low prices.” School D, Female “Children think about price.” School D, Female “If the price is affordable, we will sell.” School C, Female
Nutrition	“Interviewer: Do you sell food considering the nutritional aspect? Participant: Don’t consider it, what is important is that the food sells well.” School E, Female Interviewer: “What is taken into consideration for the products that you sell?” Participant: “The nutrition value and the food that make us full” School C, Female Participant: “The main thing we prioritize is nutritional composition first. What ingredients the food consists of healthy ingredients for children. So, if consumed it is not harmful to children and if possible, the food does not use oil, because oil is not good for health.” School B, Female	“In my opinion, like milk, it is nutritious and can improve children’s health and immune system. But “Chiki” is not nutritious because it is dangerous for a child’s brain, because of the flavoring. But the kids love it there.” School D, Female “The important thing is that the food makes us full here, the prices are cheap, there’s lots of it. They don’t think about nutrition.” School D, Female Participant: “In rural areas, many children have vitamin deficiencies, nutritional deficiencies, or what is called stunting. (Nutrition) at home is not enough, so we provide various kinds of food that contain protein, carbohydrates, iron, and so on at school.” School B, Male
Visual Appeal	“The appearance is not attractive. But if you add sugar or process it into Growol, maybe kids will want it.” School A, Female “This food is not attractive to the children” School D, Female	Participant: “Like in the shape of animals etc.” Interviewer: “What if it’s shaped just like this?” Participant: “It is not attractive, maybe the color too.” School B, Male
Profitability	“I want to sell whatever the supplier/third party sells to me; the important thing is the profit” School E “Interviewer: What are your reasons for selling several food products? Participant: Because it sells well and makes a profit” School E, Female	Interviewer: “How do you determine the selling price for children?” Participant: “If it’s packaged food like this, we take it from wholesalers. We take profits, make some profit.” School B, Male Interviewer: “Next, how do you determine the price for what has been sold?” Participant: The commercial product already has a price. For example, in 1 dozen of food, the selling price is IDR 2000,—then the profit is IDR 2000; for example, the selling price is IDR 1000,—then the profit is IDR 1000; for example, the selling price is IDR 500, then the profit is IDR 500.” School A, Female

## Data Availability

The raw data supporting the conclusions of this article will be made available by the authors on request.

## References

[1] Landscape Analysis of Overweight and Obesity in Indonesia|UNICEF Indonesia. https://www.unicef.org/indonesia/nutrition/reports/landscape-analysis-overweight-and-obesity-indonesia.

[2] Oktaviani S., Mizutani M., Nishide R., Tanimura S. (2023). Factors Associated with Overweight/Obesity of Children Aged 6–12 Years in Indonesia. BMC Pediatr..

[3] Noncommunicable Diseases: Childhood Overweight and Obesity. https://www.who.int/news-room/questions-and-answers/item/noncommunicable-diseases-childhood-overweight-and-obesity.

[4] Rachmadewi A., Soekarjo D., Maehara M., Alwi B., Mulati E., Rah J.H. (2021). School Canteens in Selected Areas in Indonesia: A Situation Analysis. Food Nutr. Bull..

[5] Hadi H., Triastanti R.K., Anggraeni D., Nurwanti E., Lewis E.C., Colon-Ramos U., Kang Y., Yamaguchi M., Gittelsohn J. (2022). The Role of the School Food Environment in Improving the Healthiness of School Canteens and Readiness to Reopen Post COVID-19 Pandemic: A Study Conducted in Indonesia. J. Public Health Res..

[6] Rachmi C.N., Jusril H., Ariawan I., Beal T., Sutrisna A. (2021). Eating Behaviour of Indonesian Adolescents: A Systematic Review of the Literature. Public Health Nutr..

[7] Ali M., Pradipta H., Syarifuddin (2022). Implementasi Manajemen Bisnis Syari’ah Untuk Peningkatan Volume Penjualan Di Kantin 1 Putera Ud. Assyarif pondok pesantren salafiyah syafi’iyah sukorejo situbondo. Al-Idarah J. Manaj. Dan Bisnis Islam.

[8] Sakai Y., Rahayu Y.Y., Araki T. (2022). Nutritional Value of Canteen Menus and Dietary Habits and Intakes of University Students in Indonesia. Nutrients.

[9] Setiawan U., Martha E., Sunardi K.S., Purnamasari A.T.P., Naftalin F., Triana E., Lidya L., Kusumawati L.R. Impact of School Policy Implementation on Snack Consumption among Primary School Students in Depok, West Java. Proceedings of the 6th International Conference on Public Health 2019.

[10] Nutrition Environment Assessment Toolkit for Schools (NEAT-S) in Indonesia|UNICEF Indonesia. https://www.unicef.org/indonesia/nutrition/reports/nutrition-environment-assessment-toolkit-schools-indonesia.

[11] da Cunha D.T., Gonçalves H.V.B., de Lima A.F.A., Martins P.A., de Rosso V.V., Stedefeldt E. (2014). Regional Food Dishes in the Brazilian National School Food Program: Acceptability and Nutritional Composition. Rev. Nutr..

[12] Addo-Preko E., Amissah J.G.N., Adjei M.Y.B. (2023). The Relevance of the Number of Categories in the Hedonic Scale to the Ghanaian Consumer in Acceptance Testing. Front. Food Sci. Technol..

[13] Meiyetriani E., Februhartanty J., Iswarawanti D.N., Sudibya A.R.P. (2019). A situational analysis of a healthy school canteen development program: Lessons learned from a selected group of primary schools in Jakarta, Indonesia. Southeast Asian J. Trop. Med. Public Health.

[14] Hadi H., Nurwanti E., Gittelsohn J., Arundhana A.I., Astiti D., West K.P., Dibley M.J. (2020). Improved Understanding of Interactions between Risk Factors for Child Obesity May Lead to Better Designed Prevention Policies and Programs in Indonesia. Nutrients.

[15] Nisak A.J., Rachmah Q., Mahmudiono T., Segalita C. (2018). Snacking Energy-Dense Food Related to Childhood Obesity. J. Nutr. Food Sci..

[16] Primasiwi C. Analysis of Variance of Ready-to-Drink Milk Brands Search Term in Indonesia. https://www.academia.edu/39762246/Analysis_of_Variance_of_Ready_to_Drink_Milk_Brands_Search_Term_in_Indonesia.

[17] Peryam D., Pilgrim F. (1957). Hedonic Scale Method of Measuring Food Preferences. Food Technol..

[18] Sijangga M., Hositanisita H., Lewis E.C., Hadi H., Matsuzaki M., Surkan P.J., Kang Y., Purnamasai S., Kurniasari Y., Gittelsohn J. (2026). Cross-sectional study of prepared foods sold in Indonesian school canteens to inform childhood obesity programs and policies. J. Nutr. Sci..

[19] Gale N.K., Heath G., Cameron E., Rashid S., Redwood S. (2013). Using the Framework Method for the Analysis of Qualitative Data in Multi-Disciplinary Health Research. BMC Med. Res. Methodol..

[20] Goldsmith L. (2021). Using Framework Analysis in Applied Qualitative Research. Qual. Rep..

[21] Maguire M., Delahunt B. (2017). Doing a Thematic Analysis: A Practical, Step-by-Step Guide for Learning and Teaching Scholars. All Irel. J. High. Educ..

[22] Hong J.-H., Cho M.-S. (2012). Acceptance of Vegetable Menus of a School Lunch Program by High School Students in Seoul and its Association with Health and Dietary Behavioral Factors. Korean J. Food Sci. Technol..

[23] Oddo V.M., Maehara M., Rah J.H. (2019). Overweight in Indonesia: An Observational Study of Trends and Risk Factors among Adults and Children. BMJ Open.

[24] Azizan N.A., Papadaki A., Su T.T., Jalaludin M.Y., Mohammadi S., Dahlui M., Nahar Azmi Mohamed M., Majid H.A. (2021). Facilitators and Barriers to Implementing Healthy School Canteen Intervention among Malaysian Adolescents: A Qualitative Study. Nutrients.

[25] Briawan D., Khomsan A., Alfiah E., Nasution Z., Putri P.A. (2023). Preference for and Consumption of Traditional and Fast Foods among Adolescentsin Indonesia. Food Res..

[26] Sree S., Palanisamy B., Sivakumar S.P. (2025). Understanding the Influencing Factors of Adolescents’ (12–17) Healthy and Unhealthy Food Choices and Experiences in Tamil Nadu, India: A Socioecological Perspective. Appetite.

[27] School Meals Coalition Press Release: Nutritious Meals Program to Reach Schools Today in Indonesia|School Meals Coalition. https://schoolmealscoalition.org/stories/press-release-nutritious-meals-program-reach-schools-today-indonesia.

